# Prediction of Response to Radiotherapy by Characterizing the Transcriptomic Features in Clinical Tumor Samples across 15 Cancer Types

**DOI:** 10.1155/2022/5443709

**Published:** 2022-05-09

**Authors:** Yu Xu, Chao Tang, Yan Wu, Ling Luo, Ying Wang, Yongzhong Wu, Xiaolong Shi

**Affiliations:** ^1^College of Bioengineering, Chongqing University, Chongqing, China; ^2^Radiation and Cancer Biology Laboratory, Radiation Oncology Center, Chongqing Key Laboratory of Translational Research for Cancer Metastasis and Individualized Treatment, Institute and Chongqing Cancer Hospital, Chongqing University Cancer Hospital and Chongqing Cancer, Chongqing 400030, China

## Abstract

**Purpose:**

Radiotherapy (RT) is one of the major cancer treatments. However, the responses to RT vary among individual patients, partly due to the differences of the status of gene expression and mutation in tumors of patients. Identification of patients who will benefit from RT will improve the efficacy of RT. However, only a few clinical biomarkers were currently used to predict RT response. Our aim is to obtain gene signatures that can be used to predict RT response by analyzing the transcriptome differences between RT responder and nonresponder groups.

**Materials and Methods:**

We obtained transcriptome data of 1664 patients treated with RT from the TCGA database across 15 cancer types. First, the genes with a significant difference between RT responder (R group) and nonresponder groups (PD group) were identified, and the top 100 genes were used to build the gene signatures. Then, we developed the predictive model based on binary logistic regression to predict patient response to RT.

**Results:**

We identified a series of differentially expressed genes between the two groups, which are involved in cell proliferation, migration, invasion, EMT, and DNA damage repair pathway. Among them, MDC1, UCP2, and RBM45 have been demonstrated to be involved in DNA damage repair and radiosensitivity. Our analysis revealed that the predictive model was highly specific for distinguishing the *R* and PD patients in different cancer types with an area under the curve (AUC) ranging from 0.772 to 0.972. It also provided a more accurate prediction than that from a single-gene signature for the overall survival (OS) of patients.

**Conclusion:**

The predictive model has a potential clinical application as a biomarker to help physicians create optimal treatment plans. Furthermore, some of the genes identified here may be directly involved in radioresistance, providing clues for further studies on the mechanism of radioresistance.

## 1. Introduction

Cancer is the leading cause of morbidity and mortality in the world, regardless of the level of human development, which is accounting for over 9.9 million deaths worldwide annually [1, 2]. Radiation therapy (RT) has played a major role in cancer therapeutics, with approximately 50% of cancer patients using RT [3]. RT mainly induces apoptosis of cancer cells by causing DNA double-strand breaks (DSBs) [4]. RT is often curative, particularly in some head and neck cancer, prostate cancer, and cervical cancer. However, the inherent radioresistance of tumor cells and the acquired radioresistance can reduce the therapeutic effect and ultimately lead to poor outcomes of patients such as tumor recurrence, metastasis, and patient death [5]. In the era of precision medicine, there is a growing need for precision radiotherapy. This requires the plans of individualized treatment by considering patients' multiple information, including the status of gene mutation and profile of gene expression, in order to achieve the best therapeutic efficacy. Radioresistance is partly due to the differences of the status of gene expression and mutation in tumors of patients. Thus, identification of patients who will benefit from RT through analyzing gene mutation or gene expression will improve the efficacy of RT.

The previous studies have demonstrated that the mutations of several genes influenced the radiosensitivity. Ataxia-telangiectasia mutated (ATM) is a central regulator of DNA damage response [6]. Mutation and inactivation of ATM can lead to increased gene instability and impaired repair of DNA double-strand breaks. Tumors harboring ATM mutation are extremely sensitive to radiation. Mutations of ATM have been found in several tumor types. Targeted next-generation sequencing has revealed an 8% incidence of ATM mutations in prostate cancer [7]. ATM is also one of the commonly mutated genes in mantle cell lymphoma (MCL) and lung adenocarcinoma [7, 8]. Recent experimental evidence demonstrated that the mutational status of ATM can be used as a biomarker for radiotherapy [9]. The correlation between KEAP1/NRF2 mutation status and radioresistance has been investigated. Keap1-Nrf2 is a master regulator of cellular response to oxidative and radiation stress [10]. The KEAP1-NRF2 pathway is involved in protection of cells from oxidative and toxic stresses. Recent studies indicated that the activating mutation of the KEAP1–NRF2 pathway induces radioresistance, and KEAP1/NRF2 mutation status is a strong predictor of RT outcome in patients with NSCLC [11].

Although the mutations of these genes could serve as predictive biomarkers for personalization of therapeutic strategies, their widespread use would be limited due to the limited number of ATM or KEAP1-NRF2 mutations carriers among cancer patients. The previous studies revealed that the difference in the status of gene expression (including lncRNA and miRNA) and DNA methylation on tumor suppressor gene may also contribute to resistance to RT. For example, radioresistance in NSCLC has been associated with overexpression of antioxidant enzymes such as Mn-superoxide dismutase (Ms-SOD) [12]. Several studies revealed many miRNAs, including miR-95 [13], miR-221, miR-222 [14], and miR-106b [15], enhanced radioresistance in cancer cells, while miR-30a [16], miR-16 [17], miR-449 [18], miR-17 [19], and miR-100 [20] enhance the radiosensitivity. The role of epigenetic modifications, especially DNA methylation, has been explored in radioresistance in malignant tumors. The changes in radiosensitivity caused by DNA methylation in the promoter region of genes associated with cell damage repair, cell proliferation, and cell cycle have been demonstrated [21]. For example, Liu et al. [22] found the promoter region of the ERCC1 gene in two radiosensitive cell lines was hypermethylated, while it was hypomethylated in the other two radioresistant cell lines. These studies provided potential biomarkers for stratification of RT patients.

In previous studies, cancer cell lines usually were used as a model for radioresistance study. We speculated that radioresistance probably involves a complex transcriptional coexpression network within tumor cells. In tumor tissue, the factors involved in the response to radiation might be more complex than in cell lines. Therefore, biomarkers obtained from the analysis of actual clinical tumor samples may be closer to clinical application. The development of high-throughput sequencing methods for mRNA (RNA-Seq) has provided a very powerful tool to analyze the transcriptome of tumors, and a huge amount of data are available [23]. In this study, we performed a large-scale analysis of transcriptomic data collected from The Cancer Genome Atlas (TCGA) from those who were treated with RT across 15 different cancer types. The gene signatures for predicting RT response were obtained by analyzing the transcriptome differences between RT responder and nonresponder groups, and the performance of gene signatures was estimated in this study.

## 2. Materials and Methods

### 2.1. Data Acquisition

The data for this study were obtained from the TCGA website (https://portal.gdc.cancer.gov/). This study downloaded 15 types of cancer clinical data and gene expression data. Fifteen types of cancers include bladder urothelial carcinoma (BLCA), invasive breast cancer (BRCA), cervical squamous cell carcinoma (CESC), esophageal cancer (ESCA), head and neck squamous cell carcinoma (HNSC), low-grade glioma (LGG), lung adenocarcinoma (LUAD), lung squamous cell carcinoma (LUSC), pancreatic cancer (PAAD), prostate adenocarcinoma (PRAD), sarcoma (SARC), cutaneous melanoma (SKCM), gastric adenocarcinoma (STAD), thyroid carcinoma (THCA), and endometrial carcinoma (UCEC). First, this study extracted the clinical treatment information and overall survival information of patients. Next, tumor tissue samples from those patients treated with radiotherapy were selected according to clinical data, and then the samples were divided into two categories according to RECIST response results: response group (including complete response and partial response) and disease progression group. Finally, 1664 tumor samples were used for analysis in this study, including 1350 response (*R*) samples and 314 progressive disease (PD) samples.

### 2.2. Expression Analysis and Differential Expression Gene Identification

Data of the mRNA profile of samples selected from the TCGA dataset were analyzed using the DESeq2 package [24] in *R* language. In the gene expression data, the genes will not be analyzed further if the read counts of these genes were less than 10 in 80% of samples. In this study, samples were divided into response (*R*) and progressive disease (PD) samples. The differentially expressed genes (DEGs) between *R* and PD samples were identified at the criteria of *p* < 0.05 and |logFC| > 1. Wald test was used to calculate the *p* value. The *t*-test was also used to calculate *p* value in this study. The top 100 genes with the significant difference were obtained for follow-up analysis. For individual tumor types, the tumor type-specific gene signature was obtained from the top 100 genes according to the *p* value of each gene expression difference in individual tumor types.

### 2.3. Binary Logistic Regression Analysis

Logistic regression analysis was used to establish a prediction model based on risk factors and used one or more explanatory variables to predict a class of response variables [25]. The standardized expression value of the top 100 genes was used as the variable; the *R* sample was used as the reference group, and the value was set to 1; the value of progressive sample was set to 0. The RT response was predicted by logistic regression modeling and quantified using a prediction index. The prediction index is the regression coefficient obtained by multiplying the expression level of the selected gene with the logistic regression model (*β*). The prediction index for each patient is as follows: predictive index = Expr gene1*∗ß* gene1 + Expr gene2*∗ß* gene2 + Expr gene3*∗ß* gene3 .......Expr geneN*∗ß* geneN + intercept.

If the prediction index is less than the threshold (e.g., < 0.5), the sample is more likely to be a progressive disease, and if the prediction index is more than the threshold, the sample is more likely to be radiosensitive.

### 2.4. K-Fold Cross Validation

In the logistic regression model, the data set was divided into a training set and a validation set. In this study, the tumor expression level data set was divided into *k* subsets (*k* = 10) by using k-fold cross validation, and the method was repeated ten times. In each repetition, one subset was randomly selected as the validation set, and the remaining *k* − 1 subset was used as the training set. The training set data were used for establishing a logistic regression model, and then validation set data were brought into the model for evaluation. In each repetition, each evaluation score was retained, while the model was discarded. After ten repetitions, the model evaluation score was used to summarize the ability of logistic regression model. AUC (area under the curve) was used to evaluate a degree or measure of separability.

### 2.5. Visualization of Differential Expression Gene

The graphics of differentially expressed genes were drawn on the basis of R. Survminer package was used to analyze and visualize the survival curve of differentially expressed genes, and log-rank test was used to calculate the *p* value. The overall survival curve was divided into high expression group and low expression group according to the gene expression level. A box plot was drawn using the ggpubr package to show the distribution of DEG in *R* and PD samples, and the *p* value was calculated using the *t*-test. The ROC curve of DEGs was drawn by using the pROC software package, and the best threshold to distinguish between *R* and PD samples was marked. Heatmap of DEGs was drawn by using pheatmap package. In the regression model, the samples were sorted from small to large according to the prediction index, and figures of prediction index were drawn by using the ggplot2 package. The best threshold point of AUC was used as a dividing line between *R* and PD samples.

### 2.6. Enrichment Pathway Analysis

Enrichment analysis of the top 100 differential genes was performed by the online tool Metascape (https://metascape.org/).

## 3. Result

### 3.1. Patient Characteristics

**Figure 1:**
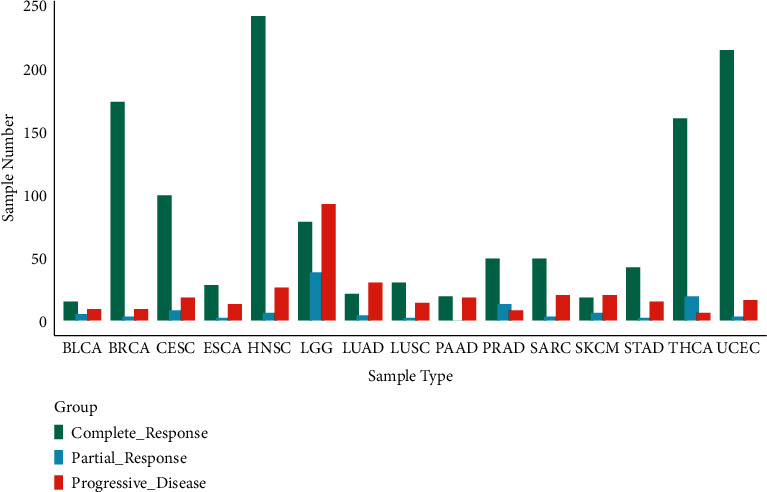
Statistical histogram of cancer types of samples and grouping.

In order to describe the transcriptome characteristics of human cancer response to radiotherapy, the transcriptome of 1664 TCGA clinical samples from 15 cancer types was analyzed, including bladder urothelial carcinoma (BLCA, *n* = 29), breast invasive carcinoma (BRCA, *n* = 185), cervical squamous cell carcinoma (CESC, *n* = 125), esophageal carcinoma (ESCA, *n* = 43), head and neck squamous cell carcinoma (HNSC, *n* = 273), low-grade glioma (LGG, *n* = 208), lung adenocarcinoma (LUAD, *n* = 55), LUSC (*n* = 46), pancreatic cancer (PAAD, *n* = 37), prostate adenocarcinoma (PRAD, *n* = 70), sarcoma (SARC, *n* = 72), cutaneous melanoma (SKCM, *n* = 59), gastric adenocarcinoma (STAD, *n* = 59), thyroid carcinoma (THCA, *n* = 44), and endometrial carcinoma (UCEC, *n* = 233). The response group (*R*) was defined as patients with a relatively good response after RT (such as complete or partial response, *n* = 1350). The progressive disease group (PD) was defined as patients with imaging progressive disease after RT (*n* = 314). The cancer types and groups of samples were shown in Figure 1.

### 3.2. Identification of Differentially Expressed Genes in *R* Group and PD Group

**Figure 2:**
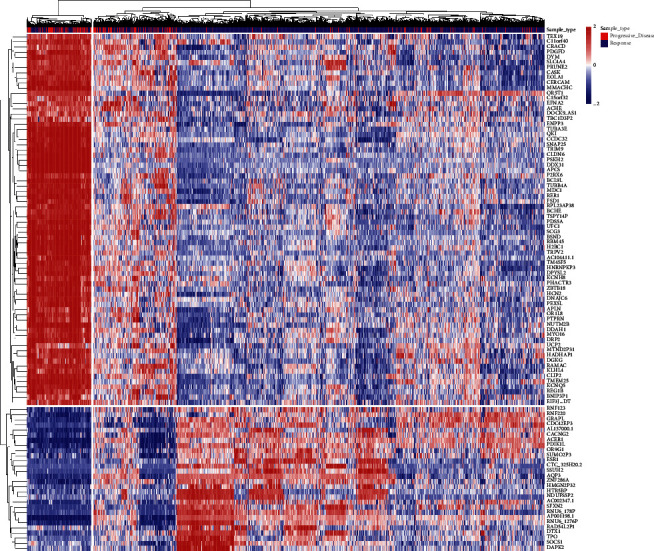
Heatmap of the top 100 differentially genes for all cancer species analyzed. Both samples and genes were clustered with average linkage.

First, according to the DEGs selection criteria | logfc | > = 1, *p* < 0.05, the differentially expressed genes between *R* and PD were identified in 15 analyzed cancer types. Then, the *t*-test is carried out using these differential genes to identify genes associated with *R* or PD (cutoff threshold *p* < 0.05). The heatmap was drawn using genes with the top 100 differential genes (Figure 2). Among the top 100 differential genes, the high expression of 72 genes and the low expression of 28 genes were highly correlated with PD samples. The functional annotation for these genes (Table S1) provides insight into the underlying biological mechanism leading to RT resistance. Genes involved in migration, cell proliferation, cell invasion, tumor metastasis, and EMT were significantly upregulated in the PD group (e.g., MDC1, UCP2, RBM45, BCL9L, P2RX6, RER1, EFNA2, CASK, CERCAM, and PTPRN). Interestingly, MDC1 has been reported as a key regulator of the DNA damage response in higher eukaryotes [26], and UCP2 and RBM45 have been implicated in RT resistance [27, 28]. Among the top 100 genes, there was a class of genes related to a ubiquitination proteasome hydrolysis system, such as RBM45, TRIM9, PTPRN, RNF123, RNF220, and DTX1. Ubiquitination is an important means of regulating target genes at the protein level. Several noncoding RNAs were also found in this study, including AC104411.1, CTC-325H20.2, AP001198.1, C15ORF32, C11ORF40, MTND2P31, RNU6-1276P, AC002347.1, RNU6-178P, HMGN2P32, and EIF3J-DT.

**Figure 3:**
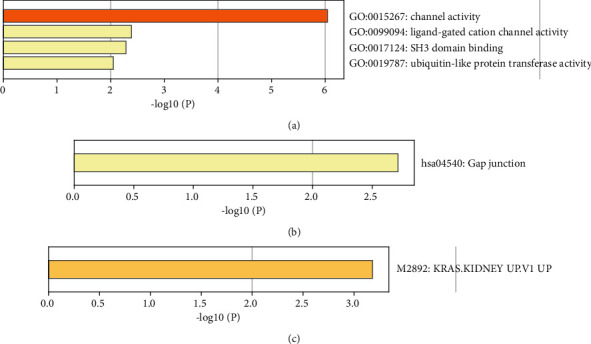
Histogram of pathway enrichment of top 100 differential genes.

GO analysis of the top 100 differential genes including biological process (BP), molecular function (MF), and oncogenic signatures (OncSig) analysis was conducted. In terms of MF, these genes are significantly enriched in channel activity, ligand-gated cation channel activity, SH3 domain binding, and ubiquitin-like protein transferase activity. BP analysis showed that the gap junction was significantly enriched. OncSig analysis showed that four genes (TRIM9, SCG3, SNAP25, and TUBB4A) were related to overexpression of KRAS. Our analysis revealed that the four genes tend to be highly expressed in PD samples. Their roles in cancer have been studied. For example, TRIM9 [29]and SCG3 [30] have been reported to promote the proliferation of cancer cell, and SNAP25 and TUBB4A were identified as potential prognostic biomarkers for prostate cancer [31] or lung adenocarcinoma [32] (Figure 3)

### 3.3. Assessing the Discriminative Power with a Single-Gene Signature

**Figure 4:**
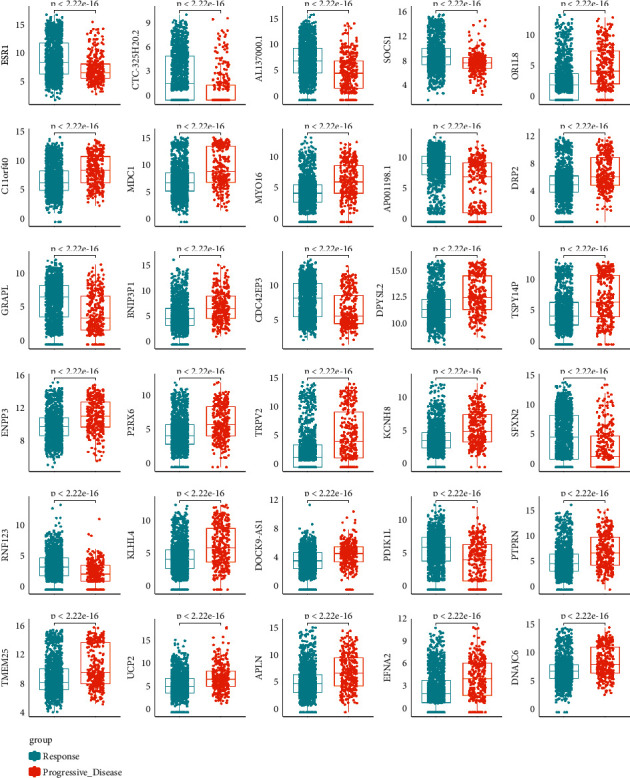
mRNA levels of differentially expressed genes between response and progressive disease tumors. The distribution of gene expression values in R or PD samples was drawn through the boxplot, and the *p* value is marked (*t*-test).

**Figure 5:**
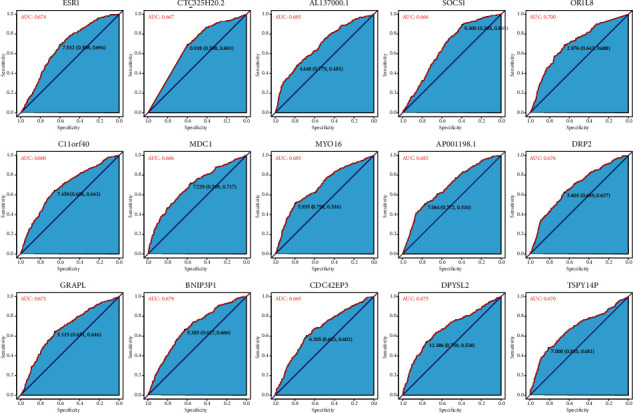
ROC curve of differentially expressed genes between response tumors and progressive disease tumors.

In this study, we identified the differentially expressed genes. We visualized the differential genes using the boxplot to estimate the discriminative power for *R* and PD samples with the single-gene signature (Figure 4). These genes show that there is a statistically significant differential expression between R and PD samples. We further used the receiver operating characteristic (ROC) curve to evaluate the specificity of single-gene signature. As shown in Figure 5, the AUC value of single-gene discrimination model was between 0.615 and 0.7, and OR1L8 gene has the highest specificity (AUC = 0.700), implying that the discriminative power of single-gene signature is not very strong. However, the expression patterns of many genes may reflect the underlying molecular mechanisms of tumor radioresistance. For example, the high expression of MDC1 will increase the ability of DNA damage repair and eventually lead to an increase in radiotherapy resistance. Therefore, we believe that the expression patterns of some of these genes still have clinical predictive power.

### 3.4. Evaluation of Survival Rate by the Single-Gene Signature

**Figure 6:**
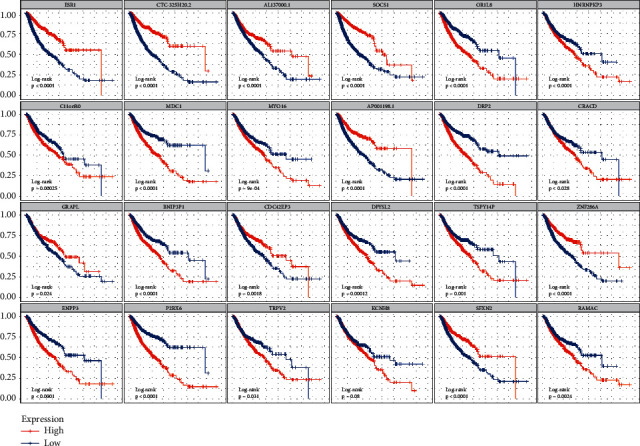
Overall survival curve of differential expression between reactive tumor and progressive disease tumor. The abscissa of the survival curve is the observation time and the ordinate is the survival rate. The median expression level of each gene was taken as the threshold, the high expression level group was higher than the median, and the low expression level group was lower than the median. The log-rank test was used to test the statistical significance of the two groups of data.

In order to investigate whether the single-gene signature has the ability to predict the survival rate of patients, we analyzed the overall survival data of the patients. The overall survival curve is shown in Figure 6. Statistically significant correlations were observed between the survival time and the expression in many chosen genes. For example, MDC1 (*p* < 0.0001), EFNA2 (*p* < 0.0001), BCL9L (*p* < 0.0001), RER1 (*p* < 0.0001), and P2RX6 (*p* < 0.0001) tended to be highly expressed in PD samples, and the overall survival rate of the high expression group was significantly lower than that of the low expression group. Conversely, ESR1 (*p* < 0.0001), RNF123 (*p* < 0.0001), DTX1 (*p* < 0.0001), and CACNG2 (*p* < 0.0001) tended to be highly expressed in the *R* sample, and the survival rate of the high expression group was significantly higher than that of the low expression group. These results revealed that *R* group patients had a better survival rate than PD patients.

### 3.5. Using Logistic Regression Model to Distinguish *R* and PD Samples

**Figure 7:**
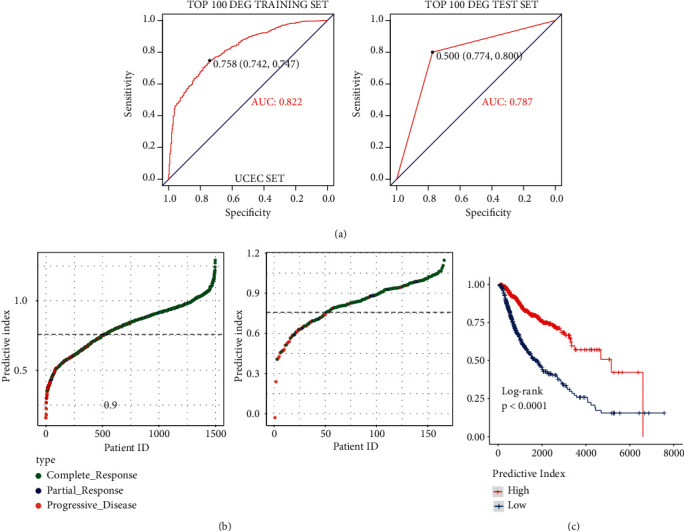
ROC curve, predictor chart, and overall survival rate chart of reactive tumor and progressive disease tumor after logistic regression. (a) ROC curve. (b) The ordinate is sorted from small to large according to the prediction index, and the threshold is 0.758. (c) Overall survival curve.

As mentioned above, the discriminative power of single-gene signature is not very strong. Therefore, we developed a binary logistic regression model to classify the samples. The prediction index was used to quantify each sample. We used 90% of the data set as the training set and 10% as the validation set. The ROC curve was drawn according to the prediction index of the regression model. Results are shown in Figure 7. The AUC value reached 0.822 and 0.787 in the training set and validation set at the best threshold score of 0.758, respectively (Figure 7(a)). Compared with the best AUC value of single-gene signature (OR1L8, AUC = 0.700), the logistic regression model has better performance for distinguishing *R* and PD samples. The performance of classifying patients by prediction index is shown by visualizing predicted and actual classifications (Figure 7(b)). The result showed that PD samples were enriched on the left side of the prediction index curve and *R* samples are enriched on the right side of the prediction index curve, implying that most of the samples can be classified correctly. We also assessed whether the prediction index can reach a better prediction for the survival rate of patients. As shown in Figure 7(c), the patients with a high prediction index had better survival than those patients with a low prediction index (*p* < 0.0001). This also implied that the RT responding patients had a better survival rate.

### 3.6. Performance of Single-Gene Signature in Different Tumor Types

**Figure 8:**
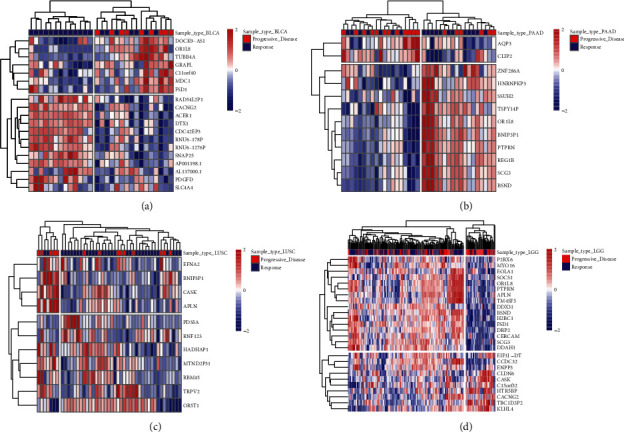
Heatmap of differentially expressed genes in different tumor types.

**Figure 9:**
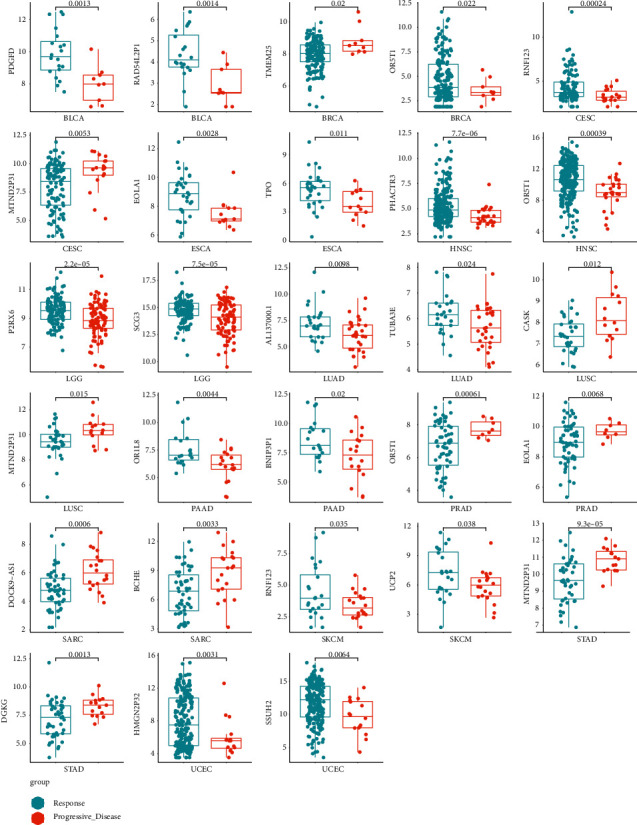
Boxplot of differentially expressed genes in different tumor types.

**Figure 10:**
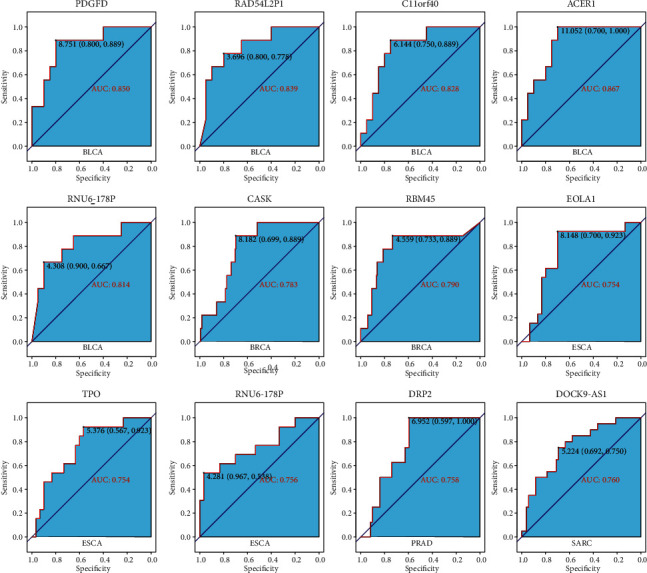
ROC map of differentially expressed genes in different tumor types.

**Figure 11:**
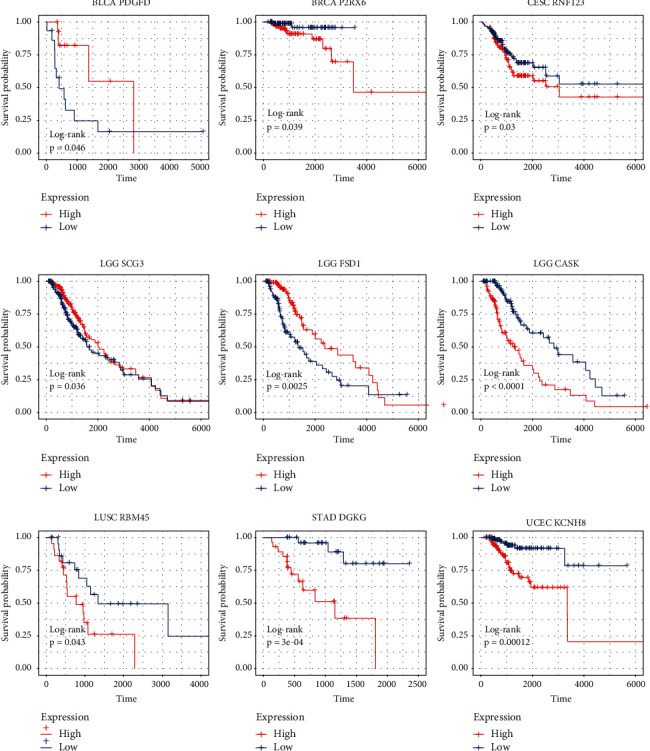
Overall survival curve of differentially expressed genes in different tumor types.

In this study, the gene signature was obtained from 1664 TCGA clinical tumor samples. The tumors derive from 15 cancer types. However, given that there may be some degree of variation in expression patterns between different tumor types, it is necessary to select the optimal subset for each tumor type from the 100-gene signature. Therefore, we analyzed the discrimination ability of each gene for *R* and PD samples in each cancer type. Genes with a *p* value of less than 0.05 (*p* value less than 0.1 in few of tumor types) were chosen to build tumor-specific gene subsets (Table S2). Cluster analysis revealed two distinct expression patterns of gene subsets in several cancer types, such as BLCA, PAAD, LUSC, and LGG (Figure 8). Many genes exhibited good ability to distinguish between *R* and PD groups (Figure 9). For example, RNF123 (*p* = 0.00024, in CESC), PHACTR3 (*p* = 7.7 *E* – 06, in HNSC), OR5T1 (*p* = 0.00039, in HNSC), P2RX6 (*p* = 2.2 *E* – 05, in LGG), and SCG3 (*p* = 7.5 *E* – 05, in LGG) were expressed higher in *R* samples, while MTND2P31 (*p* = 0.0053, in CESC), OR5T1 (*p* = 0.00061, in PRAD), EOLA1 (*p* = 0.0068, in PRAD), BCHE (*p* = 0.0033, in SARC), MTND2P31 (*p* = 9.3E − 05, in STAD), and DGKG (*p* = 0.0013, in STAD) were expressed higher in PD samples. Analysis by ROC curve also revealed several genes had high specificity of distinction (Figure 10), such as ACER1 (AUC = 0.867, in BLCA), RBM45 (AUC = 0.790, in BRCA), RNU6 − 178P (AUC = 0.756, in ESCA), and DOCK9 − AS1 (AUC = 0.760, in SARC). Several genes also exhibited the predictive power for survival of patients (Figure 11). The higher expression of P2RX6 (*p* = 0.039, in BRCA), RNF123 (*p* = 0.03, in CESC), CASK (*p* < 0.0001, in LGG), DGKG (*p* = 3E-04, in STAD), RBM45 (*p* = 0.043, in LUSC), and KCNH8 (*p* = 0.00012, in UCEC) was associated with worse survival, while the higher expression of PDGFD (*p* = 0.046, in BLCA), SCG3 (*p* = 0.036, in LGG) and FSD1 (*p* = 0.0025, in LGG) was associated with better survival.

### 3.7. Using Logistic Regression Model to Distinguish *R* and PD Samples in Different Tumor Types

**Figure 12:**
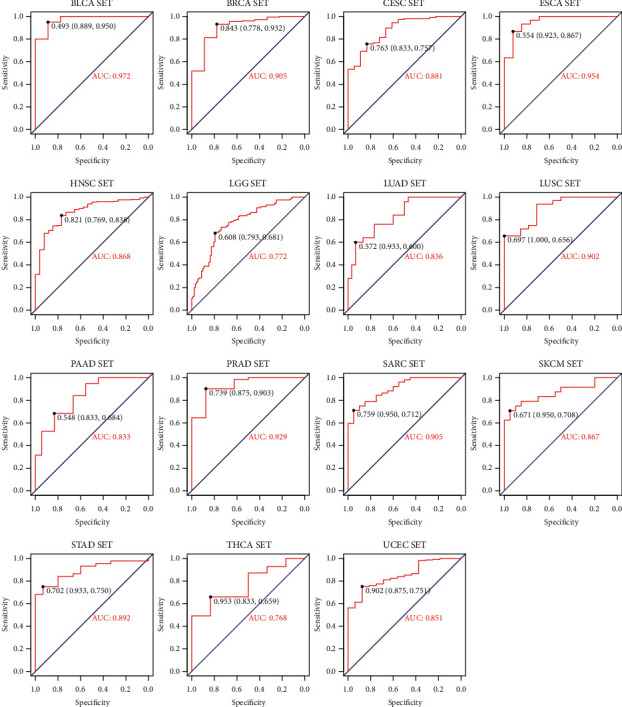
Logistic regression prediction ROC diagram of different tumor types.

**Figure 13:**
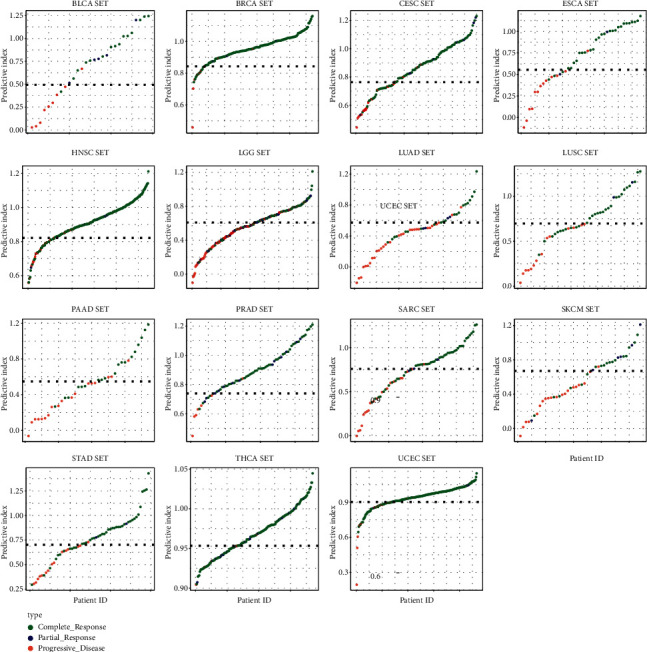
Logistic regression predictors of different tumor types.

**Figure 14:**
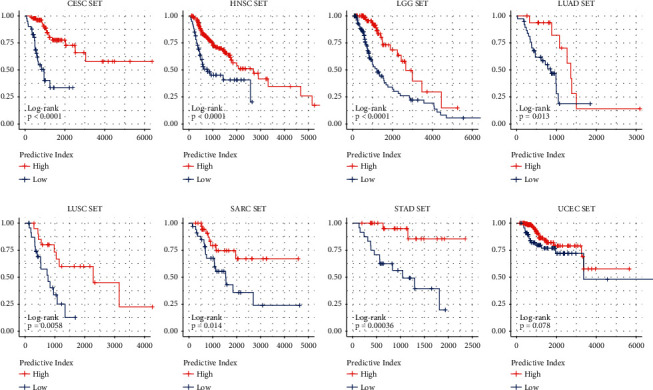
Survival curves of different tumor types.

Analysis of ROC curve revealed that the higher specificity was achieved in each cancer type by using the prediction index (Figure 12). For example, the AUC value of BLCA obtained by the prediction index was higher than from ACER1 (0.972 vs 0.867). Our results showed that most of the samples can be correctly classified in each cancer type (Figure 13). We also investigated whether the predictive model could provide better performance for the prediction of survival rate for each cancer type. As shown in Figure 14, patients with a high prediction index were associated with better survival.

## 4. Discussion

In this study, we identified the differentially expressed genes between RT responder and nonresponder. Among them, MDC1 [26], UCP2 [27], and RBM45 [28] have been demonstrated to be involved in the DNA damage pathway and radiosensitivity. MDC1 [26] was identified as a component of DNA repair complex, controlling the damage-induced cell cycle arrest checkpoint. Cells lacking MDC1 are sensitive to ionizing radiation. Our study showed that MDC1 was expressed higher in the PD group, which might lead to stronger repairability in PD patients. UCP2 [27] is a mitochondrial transporter, which can produce proton leakage on the inner membrane of mitochondria, thus uncoupling oxidative phosphorylation and ATP synthesis. The previous studies demonstrated that irradiation treatment can increase the expression level of UCP2, and silencing of UCP2 increased the radiosensitivity of HeLa cells and led to increased apoptosis, cell cycle arrest in G2/M, and mitochondrial ROS. Our results showed that the expression of UCP2 was significantly upregulated in the PD group, supporting that UCP2 has a role in radioresistance. RBM45 [28], also named DRB1, was recently found to be a FUS-interacting RBP. The previous study demonstrated that silencing of RBM45 led to a decreased efficiency in DSBs repair. Consistent with the results of the previous study, our results showed that RBM45 was expressed higher in the PD group.

Besides these three genes, several genes involving migration, cell proliferation, cell invasion, and EMT were identified in our study. For example, BCL9L [33], P2RX6 [34], RER1 [35], EFNA2 [36], CASK [37], CERCAM [38], and PTPRN [39] were demonstrated to promote EMT, metastasis, and invasion of tumor cells. Six genes involving ubiquitination proteasome process, including RBM45, TRIM9, PTPRN, RNF123, RNF220, and DTX1, were also identified. Ubiquitination plays an important role in innate and postnatal regulation of cell differentiation and cell survival. Previous studies have reported that ubiquitination-related proteins were involved in the regulation of DNA damage pathways and influenced the radiosensitivity of tumor cells [40, 41]. For example, Santra et al. found that E3 ubiquitin ligase FBXO31 mediates the degradation of cyclin D1 through ubiquitination and proteasome-mediated protein degradation pathway [40]. Knockdown of FBXO31 could prevent cells from undergoing efficient G1 arrest following *γ*-irradiation and greatly increased the sensitivity to DNA damage [41]. The differentially expressed genes also include several noncoding RNAs in our study. UFC1 is a long-chain noncoding RNA and was expressed higher in the PD group. Previous study showed that UFC1 was elevated and predicted poor prognosis of gastric cancer (GC), and knockdown of UFC1 inhibited the proliferation, migration, and invasion of GC cells [42].

Our study identified a series of genes that were differentially expressed in RT responders and nonresponders, providing useful clues for studying the molecular mechanisms of tumor radioresistance. Those genes that were overexpressed in RT nonresponders could be potential targets for radiosensitization. The prediction models and gene signatures identified here also have the potential clinical application. A targeted mRNA sequencing technology based on the gene signatures identified in this study could be developed and used to detect mRNA levels in clinical samples. By analyzing the gene signatures, patients who will benefit from RT are identified, which could reduce the number of patients receiving unnecessary treatment and greatly reduce the cost of oncology treatment.

## Data Availability

The experimental data used to support the findings of this study are available from the corresponding author upon request.

## Abbreviations

RT: Radiotherapy;
EMT: Epithelial-mesenchymal transition
AUC: Area under the curve;
DSBs: DNA double-strand breaks
RNA-Seq: High-throughput sequencing methods for mRNA;
TCGA: The Cancer Genome Atlas
BLCA: Bladder urothelial carcinoma;
BRCA: Invasive breast cancer
CESC: Cervical squamous cell carcinoma;
ESCA: Esophageal cancer
HNSC: Head and neck squamous cell carcinoma;
LGG: Low-grade glioma
LUAD: Lung adenocarcinoma;
LUSC: Lung squamous cell carcinoma
PAAD: Pancreatic cancer;
PRAD: Prostate adenocarcinoma
SARC: Sarcoma;
SKCM: Cutaneous melanoma
STAD: Gastric adenocarcinoma;
THCA: Thyroid carcinoma
UCEC: Endometrial carcinoma;
R: Response
PD: Progressive disease;
DEGs: Differentially expressed genes
ROC: Receiver operating characteristic curve;
DDR: DNA-damage response.

## Supplementary Materials

Supplementary Materials Table S1: Molecular functional annotation of top 100 differential genes in all cancer species analyzed. Table S2: DEGs used by cancer species to distinguish between R and PD samples.
