# Supplementary material for: Prediction of Response to Radiotherapy by Characterizing the Transcriptomic Features in Clinical Tumor Samples across 15 Cancer Types
Source: Comput Intell Neurosci. 2022 May 9;2022:5443709. doi: 10.1155/2022/5443709 (PMC9110128; doi:10.1155/2022/5443709)
Supplement: Supplementary Materials — Table S1: Molecular functional annotation of top 100 differential genes in all cancer species analyzed. Table S2: DEGs used by cancer species to distinguish between R and PD samples. [file 5443709.f1.docx]

TABLE S1: Molecular functional annotation of top 100 differential genes in all cancer species analyzed.

| Gene | Description | Molecular Function (GO) | mRNA expression in R group |
| --- | --- | --- | --- |
| OR1L8 | olfactory receptor family 1 subfamily L member 8 | GO:0004984 olfactory receptor activity;GO:0004930 G protein-coupled receptor activity; GO:0004888 transmembrane signaling receptor activity | Up-regulation |
| HNRNPKP3 | heterogeneous nuclear ribonucleoprotein K pseudogene 3 | processed_transcript | Up-regulation |
| C15orf32 | chromosome 15 putative open reading frame 32 | unknow | Up-regulation |
| BCHE | butyrylcholinesterase | GO:0003990 acetylcholinesterase activity;GO:0004104 cholinesterase activity;GO:0033265 choline binding | Up-regulation |
| CLDN6 | claudin 6 | GO:0001618 virus receptor activity;GO:0140272 exogenous protein binding;GO:0005198 structural molecule activity | Up-regulation |
| TEX19 | testis expressed 19 | GO:0034584 piRNA binding; GO:0061980 regulatory RNA binding ;GO:0003723 RNA binding | Up-regulation |
| C11orf40 | chromosome 11 putative open reading frame 40 | unknow | Up-regulation |
| MDC1 | mediator of DNA damage checkpoint 1 | GO:0070975 FHA domain binding;GO:0008022 protein C-terminus binding;GO:0019904 protein domain specific binding | Up-regulation |
| MYO16 | myosin XVI | GO:0003774 cytoskeletal motor activity;GO:0019903 protein phosphatase binding;GO:0019902 phosphatase binding | Up-regulation |
| DRP2 | dystrophin related protein 2 | GO:0008270 zinc ion binding; GO:0046914 transition metal ion binding;GO:0046872 metal ion binding | Up-regulation |
| CRACD | capping protein inhibiting regulator of actin dynamics | GO:0005515 protein binding; GO:0005488 binding;GO:0003674 molecular function | Up-regulation |
| TRIM9 | tripartite motif containing 9 | GO:0061630 ubiquitin protein ligase activity;GO:0061659 ubiquitin-like protein ligase activity;GO:0004842 ubiquitin-protein transferase activity | Up-regulation |
| KCNQ5 | potassium voltage-gated channel subfamily Q member 5 | GO:0005251 delayed rectifier potassium channel activity;GO:0005249 voltage-gated potassium channel activity;GO:0005267 potassium channel activity | Up-regulation |
| BNIP3P1 | BCL2 interacting protein 3 pseudogene 1 | Pseudogene | Up-regulation |
| DPYSL2 | dihydropyrimidinase like 2 | GO:0008017 microtubule binding;GO:0015631 tubulin binding;GO:0008092 cytoskeletal protein binding | Up-regulation |
| TSPY14P | testis specific protein Y-linked 14，pseudogene | pseudogene | Up-regulation |
| CLIP2 | CAP-Gly domain containing linker protein 2 | GO:0051010 microtubule plus-end binding;GO:0008017 microtubule binding;GO:0015631 tubulin binding | Up-regulation |
| ENPP3 | ectonucleotide pyrophosphatase/phosphodiesterase 3 | GO:0004528 phosphodiesterase I activity;GO:0036218 dTTP diphosphatase activity;GO:0035529 NADH pyrophosphatase activity | Up-regulation |
| P2RX6 | purinergic receptor P2X 6 | GO:0004931 extracellularly ATP-gated cation channel activity; GO:0035381 ATP-gated ion channel activity;GO:0001614 purinergic nucleotide receptor activity | Up-regulation |
| TRPV2 | transient receptor potential cation channel subfamily V member 2 | GO:0005262 calcium channel activity;GO:0015085 calcium ion transmembrane transporter activity; GO:0005261 cation channel activity | Up-regulation |
| KCNH8 | potassium voltage-gated channel subfamily H member 8 | GO:0005249 voltage-gated potassium channel activity; GO:0005267 potassium channel activity;GO:0022843 voltage-gated cation channel activity | Up-regulation |
| RAMAC | RNA guanine-7 methyltransferase activating subunit | GO:0008047 enzyme activator activity;GO:0030234 enzyme regulator activity;GO:0098772 molecular function regulator | Up-regulation |
| REG1B | regenerating family member 1 beta | GO:0070492 oligosaccharide binding;GO:0042834 peptidoglycan binding;GO:0005539 glycosaminoglycan binding | Up-regulation |
| ZBTB18 | zinc finger and BTB domain containing 18 | GO:0001227 DNA-binding transcription repressor activity，RNA polymerase II-specific;GO:0001217 DNA-binding transcription repressor activity;GO:0000978 RNA polymerase II cis-regulatory region sequence-specific DNA binding | Up-regulation |
| DGKG | diacylglycerol kinase gamma | GO:0004143 diacylglycerol kinase activity;GO:0003951 NAD+ kinase activity;GO:0016773 phosphotransferase activity，alcohol group as acceptor | Up-regulation |
| KLHL4 | kelch like family member 4 | GO:0003779 actin binding; GO:0008092 cytoskeletal protein binding;GO:0005515 protein binding | Up-regulation |
| DOCK9-AS1 | DOCK9 antisense RNA 1 | antisense | Up-regulation |
| PTPRN | protein tyrosine phosphatase receptor type N | GO:0030507 spectrin binding; GO:0044389 ubiquitin-like protein ligase binding;GO:0008134 transcription factor binding | Up-regulation |
| TUBA3E | tubulin alpha 3e | GO:0005200 structural constituent of cytoskeleton;GO:0003924 GTPase activity;GO:0005525 GTP binding | Up-regulation |
| QKI | QKI，KH domain containing RNA binding | GO:0017124 SH3 domain binding;GO:0003729 mRNA binding;GO:0019904 protein domain specific binding | Up-regulation |
| TMEM25 | transmembrane protein 25 | GO:0005515 protein binding; GO:0005488 binding;GO:0003674 molecular_function | Up-regulation |
| UCP2 | uncoupling protein 2 | GO:0017077 oxidative phosphorylation uncoupler activity;GO:0022857 transmembrane transporter activity;GO:0005215 transporter activity | Up-regulation |
| APLN | apelin | GO:0031704 apelin receptor binding;GO:0071855 neuropeptide receptor binding;GO:0005179 hormone activity | Up-regulation |
| EFNA2 | ephrin A2 | GO:0046875 ephrin receptor binding;GO:0005102 signaling receptor binding;GO:0005515 protein binding | Up-regulation |
| DNAJC6 | DnaJ heat shock protein family (Hsp40) member C6 | GO:0030276 clathrin binding;GO:0004725 protein tyrosine phosphatase activity; GO:0017124 SH3 domain binding | Up-regulation |
| SCG3 | secretogranin III | GO:0003723 RNA binding; GO:0003676 nucleic acid binding; GO:1901363 heterocyclic compound binding | Up-regulation |
| EOLA1 | endothelium and lymphocyte associated ASCH domain 1 | GO:0005515 protein binding; GO:0005488 binding;GO:0003674 molecular_function | Up-regulation |
| NUTM2B | NUT family member 2B | Diseases associated with NUTM2B include Kidney Clear Cell Sarcoma and Endometrial Stromal Sarcoma. | Up-regulation |
| RER1 | retention in endoplasmic reticulum sorting receptor 1 | GO:0033130 acetylcholine receptor binding; GO:0005102 signaling receptor binding; GO:0005515 protein binding | Up-regulation |
| SNAP25 | synaptosome associated protein 25 | GO:0017075 syntaxin-1 binding; GO:0005484 SNAP receptor activity; GO:0019905 syntaxin binding | Up-regulation |
| MTND2P31 | MT-ND2 pseudogene 31 | unknow | Up-regulation |
| PRUNE2 | prune homolog 2 with BCH domain | GO:0016462 pyrophosphatase activity; GO:0016817 hydrolase activity，acting on acid anhydrides; GO:0016818 hydrolase activity，acting on acid anhydrides，in phosphorus-containing anhydrides | Up-regulation |
| PHACTR3 | phosphatase and actin regulator 3 | GO:0008157 protein phosphatase 1 binding; GO:0004864 protein phosphatase inhibitor activity; GO:0019212 phosphatase inhibitor activity | Up-regulation |
| CASK | calcium/calmodulin dependent serine protein kinase | GO:0004385 guanylate kinase activity; GO:0042043 neurexin family protein binding; GO:0050145 nucleoside monophosphate kinase activity | Up-regulation |
| BCL9L | BCL9 like | GO:0008013 beta-catenin binding; GO:0003713 transcription coactivator activity; GO:0003712 transcription coregulator activity | Up-regulation |
| HADHAP1 | HADHA pseudogene 1 | pseudogene | Up-regulation |
| PDGFD | platelet derived growth factor D | GO:0005161 platelet-derived growth factor receptor binding; GO:0070851 growth factor receptor binding; GO:0008083 growth factor activity | Up-regulation |
| DYM | dymeclin | GO:0019899 enzyme binding; GO:0005515 protein binding; GO:0005488 binding | Up-regulation |
| SLC4A4 | solute carrier family 4 member 4 | GO:0008510 sodium:bicarbonate symporter activity;GO:0140410 solute:bicarbonate symporter activity;GO:0005452 inorganic anion exchanger activity | Up-regulation |
| PEX5L | peroxisomal biogenesis factor 5 like | GO:0005052 peroxisome matrix targeting signal-1 binding; GO:0000268 peroxisome targeting sequence binding; GO:0005221 intracellular cyclic nucleotide activated cation channel activity | Up-regulation |
| DDX31 | DEAD-box helicase 31 | GO:0003724 RNA helicase activity; GO:0008186 ATP-dependent activity，acting on RNA; GO:0004386 helicase activity | Up-regulation |
| FSD1 | fibronectin type III and SPRY domain containing 1 | GO:0008017 microtubule binding; GO:0015631 tubulin binding; GO:0008092 cytoskeletal protein binding | Up-regulation |
| RBM45 | RNA binding motif protein 45 | GO:0003723 RNA binding; GO:0003676 nucleic acid binding; GO:1901363 heterocyclic compound binding | Up-regulation |
| PDS5A | PDS5 cohesin associated factor A | GO:0005515 protein binding; GO:0005488 binding; GO:0003674 molecular_function | Up-regulation |
| UFC1 | ubiquitin-fold modifier conjugating enzyme 1 | GO:0061657 UFM1 conjugating enzyme activity; GO:0071568 UFM1 transferase activity; GO:0061650 ubiquitin-like protein conjugating enzyme activity | Up-regulation |
| DDAH1 | dimethylarginine dimethylaminohydrolase 1 | GO:0016403 dimethylargininase activity; GO:0016813 hydrolase activity，acting on carbon-nitrogen (but not peptide) bonds，in linear amidines; GO:0016597 amino acid binding | Up-regulation |
| PSKH2 | protein serine kinase H2 | GO:0106310 protein serine kinase activity; GO:0004674 protein serine/threonine kinase activity; GO:0004712 protein serine/threonine/tyrosine kinase activity | Up-regulation |
| APCS | amyloid P component，serum | GO:0001849 complement component C1q complex binding; GO:0046790 virion binding; GO:0001846 opsonin binding | Up-regulation |
| AC104411.1 | lncRNA | lncRNA | Up-regulation |
| OR5T1 | olfactory receptor family 5 subfamily T member 1 | GO:0004984 olfactory receptor activity; GO:0004930 G protein-coupled receptor activity; GO:0004888 transmembrane signaling receptor activity | Up-regulation |
| CERCAM | cerebral endothelial cell adhesion molecule | GO:0042802 identical protein binding; GO:0005515 protein binding; GO:0005488 binding | Up-regulation |
| RPL23AP38 | ribosomal protein L23a pseudogene 38 | pseudogene | Up-regulation |
| HCN2 | hyperpolarization activated cyclic nucleotide gated potassium and sodium channel 2 | GO:0005222 intracellular cAMP-activated cation channel activity; GO:0005221 intracellular cyclic nucleotide activated cation channel activity; GO:0043855 cyclic nucleotide-gated ion channel activity | Up-regulation |
| ACHE | acetylcholinesterase (Cartwright blood group) | GO:0003990 acetylcholinesterase activity; GO:0004104 cholinesterase activity; GO:0042166 acetylcholine binding | Up-regulation |
| TM4SF5 | transmembrane 4 L six family member 5 | GO:0034618 arginine binding; GO:0016597 amino acid binding; GO:0043177 organic acid binding | Up-regulation |
| H2BC1 | H2B clustered histone 1 | GO:0046982 protein heterodimerization activity; GO:0046983 protein dimerization activity; GO:0003677 DNA binding | Up-regulation |
| MMACHC | metabolism of cobalamin associated C | GO:0033787 cyanocobalamin reductase (cyanide-eliminating) activity; GO:0016723 oxidoreductase activity，acting on metal ions，NAD or NADP as acceptor; GO:0031419 cobalamin binding | Up-regulation |
| CCDC32 | coiled-coil domain containing 32 | GO:0005515 protein binding; GO:0005488 binding; GO:0003674 molecular_function | Up-regulation |
| TBC1D3P2 | TBC1 domain family member 3 pseudogene 2 | TBC1D3P2 is recurrently mutated in meningioma cell lines and is a pseudogene for TBC1D3，a known oncogene | Up-regulation |
| BSND | barttin CLCNK type accessory subunit beta | GO:0017081 chloride channel regulator activity; GO:0005254 chloride channel activity; GO:0005253 anion channel activity | Up-regulation |
| TUBB4A | tubulin beta 4A class IVa | GO:0005200 structural constituent of cytoskeleton; GO:0003924 GTPase activity; GO:0005525 GTP binding | Up-regulation |
| EIF3J-DT | EIF3J divergent transcript | lncRNA | Up-regulation |
| ESR1 | estrogen receptor 1 | GO:0030284 estrogen receptor activity; GO:0034056 estrogen response element binding; GO:0001093 TFIIB-class transcription factor binding | Down-regulation |
| CTC-325H20.2 | CTC-325H20.2 | lincRNA | Down-regulation |
| AL137000.1 | unprocessed_pseudogene | unprocessed_pseudogene | Down-regulation |
| SOCS1 | suppressor of cytokine signaling 1 | GO:0005159 insulin-like growth factor receptor binding; GO:0046935 1-phosphatidylinositol-3-kinase regulator activity; GO:0035014 phosphatidylinositol 3-kinase regulator activity | Down-regulation |
| AP001198.1 | miRNA | miRNA | Down-regulation |
| SSUH2 | ssu-2 homolog | Protein Ssu-2 Homolog；Diseases associated with SSUH2 include Rippling Muscle Disease 2 and Limb-Girdle Muscular Dystrophy Type 1C. | Down-regulation |
| RNU6-1276P | RNA，U6 small nuclear 1276，pseudogene | pseudogene | Down-regulation |
| GRAPL | GRB2 related adaptor protein like | GO:0030971 receptor tyrosine kinase binding; GO:0035591 signaling adaptor activity; GO:1990782 protein tyrosine kinase binding | Down-regulation |
| CDC42EP3 | CDC42 effector protein 3 | GO:0005519 cytoskeletal regulatory protein binding; GO:0008092 cytoskeletal protein binding; GO:0005515 protein binding | Down-regulation |
| ZNF286A | zinc finger protein 286A | GO:0000978 RNA polymerase II cis-regulatory region sequence-specific DNA binding; GO:0000987 cis-regulatory region sequence-specific DNA binding; GO:0000981 DNA-binding transcription factor activity，RNA polymerase II-specific | Down-regulation |
| ACER1 | alkaline ceramidase 1 | GO:0071633 dihydroceramidase activity; GO:0102121 ceramidase activity; GO:0017040 N-acylsphingosine amidohydrolase activity | Down-regulation |
| TPO | thyroid peroxidase | GO:0004447 iodide peroxidase activity; GO:0004601 peroxidase activity; GO:0016684 oxidoreductase activity，acting on peroxide as acceptor | Down-regulation |
| HTR5BP | 5-hydroxytryptamine receptor 5B，pseudogene | pseudogene | Down-regulation |
| SFXN2 | sideroflexin 2 | GO:0022889 serine transmembrane transporter activity; GO:0015175 neutral amino acid transmembrane transporter activity; GO:0015171 amino acid transmembrane transporter activity | Down-regulation |
| AC002347.1 | None | None | Down-regulation |
| RNF123 | ring finger protein 123 | GO:0004842 ubiquitin-protein transferase activity; GO:0019787 ubiquitin-like protein transferase activity; GO:0016740 transferase activity | Down-regulation |
| PDIK1L | PDLIM1 interacting kinase 1 like | GO:0106310 protein serine kinase activity; GO:0004674 protein serine/threonine kinase activity; GO:0004712 protein serine/threonine/tyrosine kinase activity | Down-regulation |
| OR9G1 | olfactory receptor family 9 subfamily G member 1 | GO:0004984 olfactory receptor activity; GO:0004930 G protein-coupled receptor activity; GO:0004888 transmembrane signaling receptor activity | Down-regulation |
| RNU6-178P | RNA，U6 small nuclear 178，pseudogene | pseudogene | Down-regulation |
| SUMO2P3 | SUMO2 pseudogene 3 |  | Down-regulation |
| NDUFS5P2 | NADH: ubiquinone oxidoreductase subunit S5 pseudogene 2 | pseudogene | Down-regulation |
| RAD54L2P1 | RAD54L2 pseudogene 1 | Pseudogene | Down-regulation |
| CACNG2 | calcium voltage-gated channel auxiliary subunit gamma 2 | GO:0035255 ionotropic glutamate receptor binding; GO:0035254 glutamate receptor binding; GO:0005245 voltage-gated calcium channel activity | Down-regulation |
| DTX1 | deltex E3 ubiquitin ligase 1 | GO:0005112 Notch binding; GO:0017124 SH3 domain binding; GO:0003713 transcription coactivator activity | Down-regulation |
| DAPK2 | death associated protein kinase 2 | GO:0005516 calmodulin binding; GO:0106310 protein serine kinase activity; GO:0004674 protein serine/threonine kinase activity | Down-regulation |
| RNF220 | ring finger protein 220 | GO:0061630 ubiquitin protein ligase activity; GO:0061659 ubiquitin-like protein ligase activity; GO:0004842 ubiquitin-protein transferase activity | Down-regulation |
| HMGN2P32 | high mobility group nucleosomal binding domain 2 pseudogene 32 | processed_pseudogene | Down-regulation |
| AQP3 | aquaporin 3 (Gill blood group) | GO:0015254 glycerol channel activity; GO:0015204 urea transmembrane transporter activity;GO:0015168 glycerol transmembrane transporter activity | Down-regulation |

TABLE S2: DEGs used by cancer species to distinguish between R and PD samples.

| Cancer type | Gene |
| --- | --- |
| BLCA | PDGFD，RAD54L2P1，C11orf40，ACER1，RNU6-178P，RNU6-1276P，OR1L8，DOCK9-AS1，AL137000.1，DTX1，CDC42EP3，SNAP25，MDC1，TUBB4A，GRAPL，AP001198.1，SLC4A4，CACNG2，FSD1 |
| BRCA | TMEM25，OR5T1，RNF123，CASK，P2RX6，TEX19，EOLA1，AC002347.1，DAPK2，RBM45 |
| CESC | RNF123，MTND2P31，TBC1D3P2，HNRNPKP3，SUMO2P3，CERCAM，DDX31，DNAJC6，CASK，PRUNE2，REG1B，CRACD，HADHAP1，DOCK9-AS1 |
| ESCA | EOLA1，TPO，RNU6-178P，DPYSL2，TRPV2，MMACHC，DOCK9-AS1，PDS5A，BCHE，HNRNPKP3 |
| HNSC | PHACTR3，OR5T1，EIF3J-DT，AQP3，KCNQ5，TEX19，ZNF286A，PDGFD，OR1L8，P2RX6，NUTM2B，MMACHC，CRACD，GRAPL，TPO，APCS，TUBB4A，TMEM25，PEX5L |
| LGG | P2RX6，SCG3，FSD1，CASK，CERCAM，DRP2，C15orf32，CLDN6，OR1L8，BSND，DDAH1，EOLA1，SOCS1，PTPRN，TBC1D3P2，CCDC32，DDX31，CACNG2，KLHL4，H2BC1，HTR5BP，MYO16，EIF3J-DT，APLN，TM4SF5，ENPP3 |
| LUAD | AL137000.1，TUBA3E，UCP2，MYO16，SFXN2，TRIM9，PRUNE2，BNIP3P1，SCG3 |
| LUSC | CASK，MTND2P31，PDS5A，RBM45，APLN，TRPV2，RNF123，HADHAP1，EFNA2，BNIP3P1，OR5T1 |
| PAAD | OR1L8，BNIP3P1，PTPRN，SSUH2，SCG3，AQP3，REG1B，BSND，ZNF286A |
| PRAD | OR5T1，EOLA1，DRP2，ACER1，ESR1，DDAH1，TM4SF5，BNIP3P1，SNAP25，TEX19，CACNG2，RER1，C15orf32，PDGFD |
| SARC | DOCK9-AS1，BCHE，EOLA1，HADHAP1，SNAP25，TUBA3E，EIF3J-DT，KCNH8，AL137000.1，MMACHC，TRIM9，PRUNE2，MDC1，OR1L8，RNF220，TUBB4A，SCG3 |
| SKCM | RNF123，UCP2，BSND，BCL9L，GRAPL，REG1B，TRIM9 |
| STAD | MTND2P31，DGKG，RNF220，DTX1，SUMO2P3，ACHE，MYO16，TSPY14P，TMEM25，BCHE，QKI |
| THCA | SCG3，ACHE，BCL9L |
| UCEC | HMGN2P32，SSUH2，BNIP3P1，ESR1，MMACHC，KCNH8，PTPRN，TEX19，ZNF286A，HADHAP1，FSD1，SCG3，EOLA1，DNAJC6，NUTM2B，RNU6-1276P，PHACTR3，PDIK1L，TRIM9，DOCK9-AS1，REG1B，C11orf40，TUBB4A，RNF123，ACER1，HNRNPKP3 |
| ALL | ESR1，CTC-325H20.2，AL137000.1，SOCS1，OR1L8，HNRNPKP3，C15orf32，BCHE，CLDN6，TEX19，C11orf40，MDC1，MYO16，AP001198.1，DRP2，CRACD，TRIM9，KCNQ5，SSUH2，RNU6-1276P，GRAPL，BNIP3P1，CDC42EP3，DPYSL2，TSPY14P，ZNF286A，ACER1，TPO，CLIP2，HTR5BP，ENPP3，P2RX6，TRPV2，KCNH8，SFXN2，RAMAC，REG1B，AC002347.1，ZBTB18，DGKG，RNF123，KLHL4，DOCK9-AS1，PDIK1L，PTPRN，OR9G1，RNU6-178P，SUMO2P3，TUBA3E，QKI，TMEM25，UCP2，APLN，EFNA2，DNAJC6，SCG3，EOLA1，NDUFS5P2，RAD54L2P1，NUTM2B，RER1，SNAP25，CACNG2，MTND2P31，PRUNE2，PHACTR3，CASK，BCL9L，HADHAP1，PDGFD，DTX1，DYM，SLC4A4，DAPK2，PEX5L，DDX31，FSD1，RBM45，PDS5A，UFC1，DDAH1，PSKH2，RNF220，APCS，AC104411.1，OR5T1，CERCAM，RPL23AP38，HMGN2P32，HCN2，ACHE，TM4SF5，H2BC1，MMACHC，CCDC32，TBC1D3P2，BSND，TUBB4A，EIF3J-DT，AQP3 |
